# A Multi-Disciplinary Approach in the Management of Endo-Perio Lesions: A 4-year Follow-Up Case Report

**DOI:** 10.1155/2023/3024231

**Published:** 2023-01-23

**Authors:** Stephani Dwiyanti

**Affiliations:** Department of Dental Medicine, School of Medicine and Health Sciences, Atma Jaya Catholic University of Indonesia, Jakarta, Indonesia

## Abstract

*Summary*. This case report discusses the multi-disciplinary approach and long-term follow-up of a 66-year-old male who suffered a combined endodontic–periodontal lesion (EPL). As EPL is uncommon in daily practice and dentists' knowledge and awareness of EPL is quite low, this case becomes of high interest and value to document and research. The objective of this study is to present the diagnosis, multi-disciplinary approach, and long-term follow-up of compromised teeth with EPL. It highlights the importance of the identification and elimination of all causative factors as well as the correct treatment sequence to achieve a predictable outcome. The patient was referred to the periodontist after multiple unsuccessful attempts by his previous dentist. He complained of recurrent dull pain and abscess on his upper left tooth (tooth #26) that had been present for the past three years. A diagnosis of combined EPL was obtained after thorough anamnesis, clinical evaluation, and radiographic examination. The clinician identified several predisposing factors, such as plaque, trauma from occlusion, and excessive force on tooth #26 due to incorrect denture design. Treatment involved multiple dental specialties. At the periodontist, the patient underwent scaling, root planning, and removal of overhanging part of the restoration. At the endodontist, root canal treatment (RCT) was completed. Two months after RCT, a periodontal regenerative procedure was done. The defect was filled with a combination of allograft/alloplastic bone graft and covered with a barrier membrane. Upon healing, the prosthodontist did the final restoration of fiber post with a metal crown on tooth #26 and constructed a new denture with a periodontal-friendly design. A follow-up at five months and four years showed excellent results. The patient was symptom-free, and tooth #26 showed no periodontal inflammation. Radiographic examination showed a good bone fill at the defect. Supportive periodontal therapy should be emphasized to achieve the long-term success of EPL.

## 1. Introduction

The pulp and periodontal tissue are biological complexes that are closely related uniquely [1]. Many physiologic communication paths exist, such as through the exposed dentinal tubules, lateral and accessory canals, or apical foramen [1, 2]. Those tissues can be infected individually or combined, and when both systems are involved, they are termed endodontic–periodontal lesions (EPLs) [1].

For many years, the EPL has always been a clinical dilemma. This lesion is very complex and can have varied pathogenesis [3]. It describes a pathologic pathway between a tooth's pulp and periodontal tissue that several etiologies can trigger [3]. The EPL can be caused by caries, trauma, restorative procedures, chemical irritation, or severe thermal stimulation that affects the pulp and, secondarily, the periodontium [3, 4]. In this scenario, an inflammatory lesion on the pulp causes localized edema and increases intrapulpal pressure, leading to cell death [4]. An increased intrapulpal pressure may force the toxic agents through the apical foramen, lateral and accessory canals, or dentinal tubules [4]. This results in retrograde periodontitis.

Another possible pathogenesis of EPL is the destruction of the periodontium that secondarily affects the root canals [3, 4]. Plaque and calculus are responsible for this periodontal lesion [4]. Inflammatory mediators destroy gingival connective tissue, periodontal ligament, and alveolar bone [4]. There is also a loss of the outer cementoblast layer, resulting in a shallow resorptive lesion of the cementum [4]. The exposure of dentinal tubules on the missing parts of the cementum will allow the bacterial invasion to the tubules and increase the likelihood of cumulative damage to the pulp, leading to retrograde pulpitis [4]. Periodontal disease can progress apically and involve the apical foramen, causing pulpal necrosis [2, 4].

The pathogenesis of a true-combined lesion is similar to that of the primary endodontic and periodontal lesion. The merging of two independent lesions forms it: the periapical lesion originating from the necrosis pulp and the periodontal lesion progressing apically [3, 4]. Lastly, EPL can also develop through nonphysiological pathways, such as iatrogenic root canal perforations and vertical root fractures (VRFs) [1, 2]. Through these pathways, organisms can spread from the infected pulp to the healthy periodontium or from the periodontal lesion to a vital pulp [1, 2].

EPL's etiology must be correctly identified to achieve a successful treatment plan. Regardless of their area of specialization, clinicians should have a thorough understanding and scientific knowledge of this lesion [5]. Unfortunately, a study by Çirakoğlu and Karayürek found that dentists' level of knowledge and awareness regarding EPL is quite low [1]. They evaluated the awareness of dentists about EPL according to their area of expertise and discovered that endodontists and periodontists were significantly more aware of EPL compared with other expert dentists [1]. It was also observed that general practitioners did not have significant awareness regarding EPL [1]. It will be a problem because a correct treatment sequence and a multi-disciplinary approach should be involved to achieve the best outcome for EPL [5].

Before doing any advanced restorative or surgical work to treat EPL, the prognosis of the tooth must be weighed carefully. Extraction should be considered if the patient has a low motivation and refuse lengthy, invasive, and expensive treatment [6]. If the tooth is non-functional or adequate root canal treatment (RCT) is not possible, there is no point in keeping the tooth [6]. In most cases, root canal therapy should be performed before periodontal treatment [6]. Periodontal treatment can be in the form of bone grafting and guided tissue regeneration, hemisection, or root amputation [6].

A study by Prashaanthi et al. in an institution discovered that 17.3% of the study population had an endo-perio lesion, and its prevalence was higher among males (12.7%) and the age group of 31–40 years (5.3%) [7]. Unfortunately, there is no national data regarding the prevalence of endo-perio lesions in Indonesia. There is also limited study about this lesion in the Indonesian population, which encourages the author to explore the topic further.

This study aims to present the diagnosis, multi-disciplinary approach, and long-term follow-up of compromised teeth with endo-perio lesions. It highlights the importance of the identification and elimination of all causative factors as well as the correct treatment sequence to achieve a predictable outcome.

## 2. Case Presentation

### 2.1. Diagnosis and Etiology

A 66-year-old male patient with no medical condition complained of recurrent dull pain and an abscess on his upper left tooth that had been present for the past three years. When the pain and abscess occurred, he would visit the dentist to seek treatment. His previous dentist would do a curettage on the affected area and prescribe the anti-inflammatory agent Diclofenac Potassium. Nevertheless, the pain and abscess would eventually return within three months. He recalled that he had a filling on the affected tooth, which was done more than ten years ago. He had also been using removable dentures for the last five years with good maintenance. His last visit to the dentist was one week ago, in which the dentist drained his abscess and prescribed 500 mg Amoxicillin and 500 mg Metronidazole for five days each, and 50 mg Diclofenac Potassium. He was also told to rinse with warm salt water three times a day, and was referred to a periodontist.

**Figure 1:**
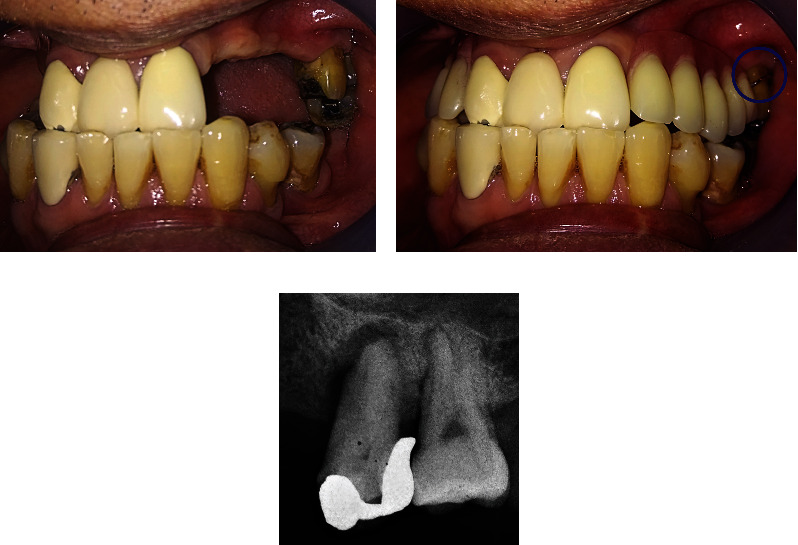
Initial condition. (a) Intraoral condition of gingival region tooth #26 showed recession without visible abscess. (b) Intraoral condition when the patient wore denture with a single clasp on tooth #26 (shown in blue circle). (c) Initial radiograph condition. Overhanging restoration on the distal part of the crown. Radiolucency surrounded the mesial and apical parts of the roots.

No abnormality was found upon extraoral examination of the head and neck. On intraoral examination, no abscess was evident on the gingival region of the upper left maxillary first molar (tooth #26), as shown in Figure 1(a). Tooth #26 had an amalgam filling on the mesial, occlusal, and distal parts and exhibited grade II tooth mobility. The patient had no response on the pulp vitality test, but he felt discomfort upon percussion. Periodontal examination showed a pocket of 14 and 7 mm on the mesial and distal aspects of tooth #26, respectively (Table 1). A pocket of 5 mm was found on the mesial of tooth #27 (Table 1). The gingiva bled upon probing and showed a recession of 2–3 mm. Keratinized tissue width was approximately 2–3 mm. Intraoral examination revealed interferences between teeth #26 and #38 upon articulation towards the working, non-working, and anterior sides. The patient had good oral hygiene even though he had multiple missing teeth. He wore an upper removable partial denture to replace teeth #13–#17 and #22–#25, with only one half-Jackson clasp anchored on tooth #26 (Figure 1(b)). Preoperative periapical radiograph revealed a mesio–occluso–distal restoration reaching the dentin on the coronal part, with evidence of overhanging on the distal part. Tooth #26 had convergent roots. An area of radiolucency surrounded the mesial and apical parts of the roots with a thickness of approximately 2 mm (Figure 1(c)).

The diagnosis of combined EPL was obtained according to the 2017 World Workshop on the Classification of Periodontal and Peri-implant Diseases and Conditions. Predisposing factors identified were plaque, trauma from occlusion (TFO) due to interferences of teeth #26 and #38, and excessive force on tooth #26 due to using a single clasp on the denture design. The case has a guarded prognosis, and the patient and clinician decided to save the tooth.

The differential diagnosis for tooth #26 was VRF because it presented with recurrent dull pain, abscess, and slight tooth mobility [8]. There were also isolated deep pockets, especially on the mesial part, with generally much shallower pockets on other parts, which was a characteristic of VRF [8]. Unfortunately, cone beam computed tomography was not performed because it was not available at the dental office, and the patient refused the examination due to cost. Eventually, a differential diagnosis of VRF was omitted because the tooth responded well to endodontic treatment.

### 2.2. Treatment Alternatives

The first treatment alternative offered to the patient was the extraction of tooth #26. It was the fastest and less complicated way to resolve the infection. The missing tooth could be replaced with a removable denture or dental implant. However, the patient refused another tooth extraction because he had lost most of his upper teeth. He also refused a dental implant due to financial issues.

Another possible alternative is using probiotics and antibiotics as an adjunct to scaling and root planning (SRP) [9]. During drainage of the abscess before meeting the periodontist, the patient was already given Amoxicillin and Metronidazole. As a result, the periodontist decided not to prescribe another antibiotic. Meanwhile, probiotics were not officially distributed in Indonesia during the patient's treatment. Therefore, it was not given as an adjunctive therapy.

The last alternative is using ozone and photobiomodulation, in addition, to SRP to treat periodontal disease [10]. According to Scribante et al., the use of those adjuvant therapies had significantly reduced values of probing pocket depth, plaque index, and bleeding on probing (BoP). Unfortunately, such modalities were unavailable in the office and consequently not offered to the patient as an alternative.

### 2.3. Treatment Progress

The first phase of the therapy consisted of scaling, root planning, and removal of overhanging margin on the distal part of tooth #26. The patient was then referred to an endodontist for RCT, performed using ProTaper Universal System (Dentsply Sirona, Charlotte, NC, USA). Ultracal™ XS Calcium Hydroxide Paste medication was used between appointments (Ultradent, South Jordan, UT, USA). The tooth was obturated with a single cone obturation technique of gutta-percha and AH Plus sealer (Dentsply Sirona).

**Figure 2:**
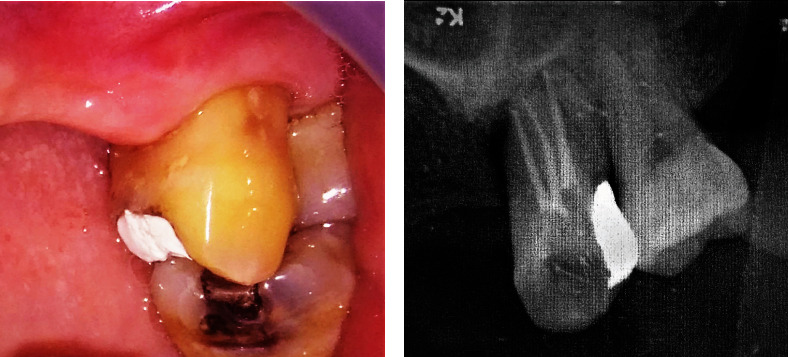
Clinical and radiographic examination of tooth #26 after root canal treatment. (a) Temporary filling covered the mesio-occlusal part of crown tooth #26. Gingiva looked normal. (b) Radiographic examination showed obturation of root canals of tooth #26 with decreased radiolucency on the apical part of the tooth.

Upon completion of the treatment, the patient was referred back to the periodontist with the suggestion of putting a final restoration of the dowel crown after periodontal flap surgery. The patient admitted that he no longer experienced pain and abscess on tooth #26. Clinical examination showed temporary restoration on tooth #26. Gingiva around the tooth looked normal, but it bled upon probing. There was a significant pocket reduction on tooth #26, with 6 and 5 mm on the mesial and distal pockets of the tooth, respectively (Figure 2(a)). The tooth still exhibited grade II tooth mobility. Meanwhile, the mesial pocket of tooth #27 was 4 mm. Radiographic examination showed a significant decrease in radiolucency, especially around the apical part of tooth #26. However, radiolucency on the mesial aspect of the root persisted (Figure 2(b)).

**Figure 3:**
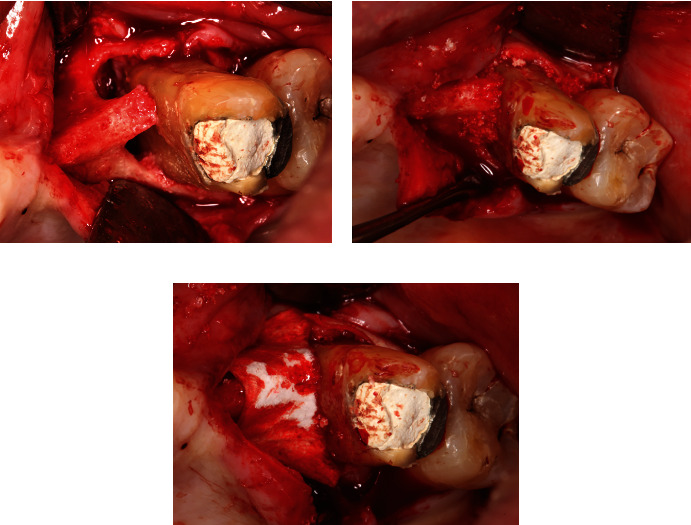
Guided tissue regeneration (GTR). (a) Infrabony defect on the mesial and buccal part of tooth #26, with some bone holding the middle third of the mesial root. (b) Packing of defect with a combination of allograft/alloplastic bone graft. (c) Pericardium membrane was placed over the defect.

A periodontal regenerative procedure was planned two months following RCT. A full-thickness mucoperiosteal flap was elevated from the edentulous side of tooth #25 up to the distal part of tooth #27. Infrabony defect with a depth of 14 mm was found on the mesial aspect of tooth #26, which extended to the buccal area between tooth #26 and tooth #27 (Figure 3(a)). The defect was filled with bone graft material freeze-dried bone allograft (Batan, Jakarta, DKI Jakarta, Indonesia) and alloplastic (Ossifi, Kansas, MO, USA; Figure 3(b)). A barrier membrane (Batan, Jakarta) was placed over the graft, and the flap was sutured with 5-0 nylon sutures (Ailee, Sasang-gu, Busan, South Korea; Figure 3(c)). Periodontal dressing (GC America, Alsip, IL, USA) was applied on top of the flap, and post-operative instructions were given. Medications prescribed to the patients were Amoxicillin-clavulanate 500 mg (Klavamox, Jakarta, DKI Jakarta, Indonesia) and Metronidazole 500 mg for five days (Kimia Farma, Jakarta, DKI Jakarta, Indonesia), Diclofenac Potassium 50 mg (Hexpharm Jaya, Jakarta, DKI Jakarta, Indonesia), and chlorhexidine gluconate 0.2% as a mouth rinse (Minorock Mandiri, Depok, West Java, Indonesia).

**Figure 4:**
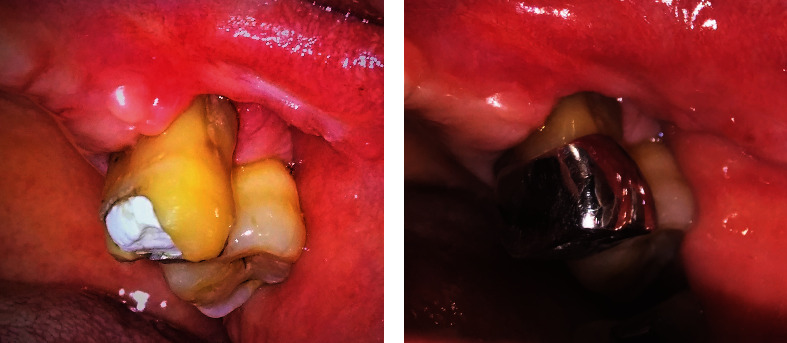
Post-operative condition: (a) One month post-operative: gingival recession was evident, especially on the buccal of tooth #26. (b) Five months post-operative: temporary restoration has been replaced by a metal crown. The gingival margin migrated toward the coronal direction. Scar tissue on mesial of tooth #26 was less noticeable.

**Figure 5:**
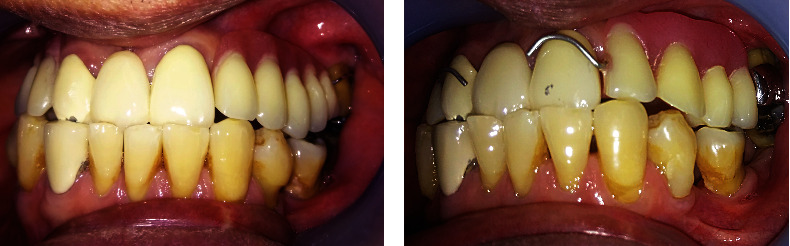
Comparison of maxillary denture design: (a) Faulty design of the previous denture with only a single clasp on tooth #26. (b) New design with multiple clasps for better distribution of load.

Post-operative healing was satisfactory, with minimal discomfort. A one-month follow-up showed a gingival recession in the buccal area of tooth #26, which was typical and expected (Figure 4(a)). As the patient did not exhibit any more symptoms on the tooth and gingiva, he was referred to a prosthodontist for final endodontic restoration using fiber post and crown. A metal crown was installed on tooth #26. The patient was suggested to change his denture. Instead of using a single clasp (Figure 5(a)), a new acrylic denture was installed with multiple clasps on teeth #12, #21, and #27 (Figure 5(b)).

### 2.4. Treatment Results

Five-month post-operative follow-up showed the good condition of the tooth. The tooth exhibited minimum mobility, the gingiva had entirely healed with minimum scar tissue, and creeping attachment was noticeable on the buccal area (Figure 4(b)) compared with the previous position (Figure 4(a)). Only mesiopalatal tooth #26 showed a pocket depth of 5 mm, whereas the rest ranged from 1 to 3 mm. Nevertheless, no bleeding/gingival inflammation was found. The patient was encouraged to come for a recall visit every three months.

Four years later, the patient came back for re-evaluation. He was satisfied because he did not experience recurring pain or abscess, and his new denture was comfortable and functional. He admitted that he had not done any follow-up or cleaning for these past four years. His oral hygiene was excellent.

**Figure 6:**
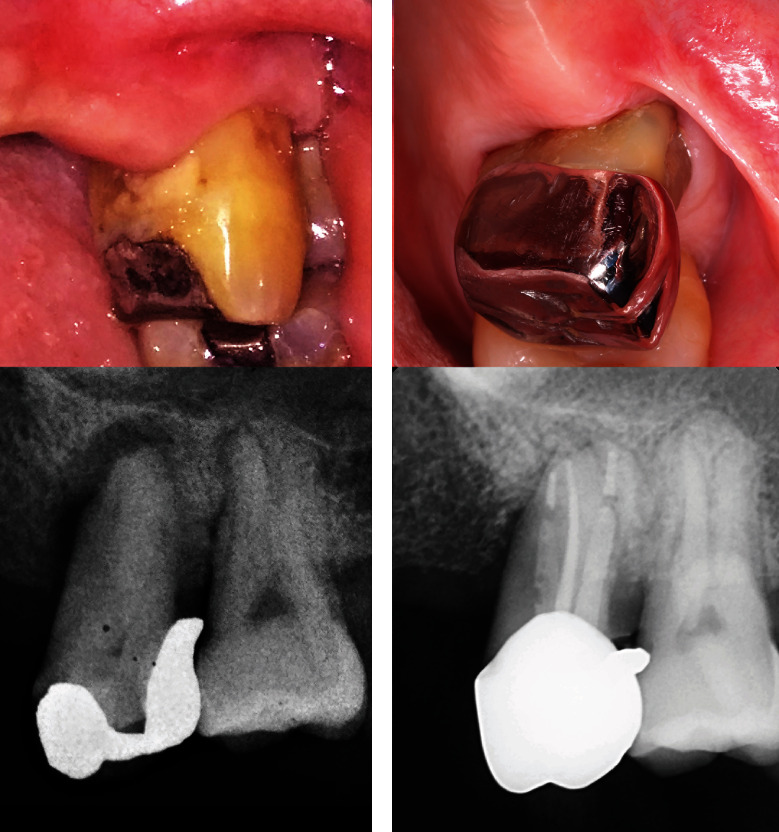
Comparison between (a) initial and (b) final clinical and radiographic condition.

Tooth #26 showed minimal mobility. The frenulum at the mesial aspect of tooth #26 was noticeably high, yet no significant recession was found compared with the initial condition (Figure 6). The occlusal function was also evaluated to check for any premature contact and blocking, and no abnormalities were found. When compared with the initial radiographic examination, significant improvement was observed. There was an increase in radiopacities, particularly on the apical and mesial parts of teeth #26 and #27. The patient was encouraged to return for routine follow-ups every 3–6 months to maintain his oral condition.

## 3. Discussion

The diagnosis of EPL is often difficult because endodontic and periodontal lesions are usually studied as separate entities, and each primary lesion may sometimes mimic the clinical characteristic of the other [11]. Accurate lesion classification is the first step in aiding clinicians in planning the most appropriate treatment strategy [12, 13]. To do this, good history, clinical evaluation, and radiographic examination must be performed. Besides, additional diagnostic tests, such as vitality tests, percussion, mobility, and periodontal probing should be done [12, 13]. These basic tests differentiate between pulpal and periodontal disease [12, 13].

Some clinicians tend to skip certain diagnostic steps, especially probing measurement. This case report intends to remind clinicians to always follow a step-by-step examination. Understanding the disease's complexity helps keep the treatment options open rather than going straight for extraction. While other studies mostly explain endodontic and surgical periodontal treatment (bone grafting and GTR, root amputation, and hemisection/bicuspidization) in treating EPL, this case report wants to guide the reader to consider all other predisposing factors for EPL, which are sometimes overlooked [5, 6, 11]. According to Anand et al., the ideal therapeutic sequence for a truly combined lesion is root canal therapy, review after 2–3 months, and perform periodontal therapy if the lesion is not showing signs of resolving [6]. The author of the current study suggests adding more detailed periodontal treatment that needs to be considered for EPL, such as splinting, removing occlusal interferences, correcting overhanging restoration, and creating a periodontal-friendly prosthetic design. Addressing these minor details play a part in the success of EPL treatment.

Clinical and radiographic signs of TFO were found in tooth #26. Ongoing TFO leads to funnel-shaped widening of the crestal part of the periodontal ligament and resorption of the adjacent bone, leading to angular bone defect, weakened tooth support, and tooth mobility [14]. Radiographically, there was vertical rather than horizontal destruction of the interdental septum [14]. Clinical findings during flap operation further supported it: a vertical bone loss on the mesial of tooth #26. In the case of the unknown cause of recurrent periodontal/periapical abscess, the author suggests always checking for occlusal prematurities and interferences.

The patient's initial dentist diagnosis was incorrect, as the dentist addressed only the periodontal issue. The dentist probably did not consider endodontic origin because the restoration on tooth #26 was far from the pulp. A pivotal finding that suggested combined EPL, not periodontal lesion only as previously thought, was that the abscess kept coming back after curettage alone but did not reappear after endodontic treatment [6]. Performing periodontal treatment alone in the presence of a truly combined lesion may lead to initial healing of the periodontal pocket. Still, it will break down rapidly if pulpal infection is not eliminated [6]. The author deduced that the abscess reappeared because closed curettage alone would lead to new attachment formation only on the coronal portion of the pocket. Thereby sealing off that portion, eventually leading to the reformation of periodontal abscess on the apical part.

The author used the trimeric model of periodontal treatment planning [15]. SRP removed plaque and calculus while polishing the distal part of amalgam restoration of tooth #26 eliminated iatrogenic factors. In the case of primary periodontal disease with secondary endodontic involvement and true combined endodontic–periodontal disease, both endodontic and periodontal therapies are needed [6]. Usually, RCT should be performed before periodontal therapy, as periodontal lesions sometimes resolve after successful root canal therapy, whereas the opposite is seldom true [6, 11].

The patient then entered the surgical phase (phase II) in the form of guided tissue regeneration. Bashutski et al. published a decision-making model for guided tissue regeneration [16]. According to their study, other than controlling systemic and environmental factors, it was essential to establish a conducive local environment for periodontal regeneration [16]. Some local factors to be considered are tooth morphology, gingival condition, tooth mobility, and the number of tooth roots [16]. GTR on tooth #26 was ideal because it had no local factors that prevent connective tissue attachment, such as crown or restoration with apical margin, enamel pearls, or enamel cervical projection. A thick gingival biotype with sufficient keratinized tissue width of 2 mm could help in primary wound closure and angiogenesis [17]. Lastly, the tooth had multiple roots but no furcation involvement [16].

The metal crown was chosen in the restorative phase (phase III) because of its strength, durability, and lower cost. The margin of the crown was placed supragingival after considering several factors. First, the tooth involved was a posterior tooth with no aesthetic concern. Second, a supragingival margin was linked to better periodontal health and hygiene [18]. Nayer et al. investigated the clinical effect of crown margin position and crown material on periodontal health [18]. The study found that teeth restored with supragingival margin scored better in all indexes than those with subgingival margins. Nevertheless, there was no difference between all-ceramic and porcelain fused to metal crowns on periodontal health [18].

Periodontal maintenance should be performed at selected time intervals to assist periodontal patients in maintaining oral health and decreasing disease recurrence [15]. Farooqi et al. investigated the most appropriate time intervals for periodontal maintenance of patients previously treated with chronic periodontal disease. They concluded that the “one size fits all” recommendation (every two weeks, three months, and six months) was questionable [19]. Lang and Tonetti formulated a periodontal risk assessment tool, which assesses six major factors that contribute to periodontal diseases, such as the prevalence of BoP, residual periodontal pockets, and tooth loss, estimation of periodontal support loss concerning patient's age, systemic condition, and environmental factors [20]. The patient in this report has a medium periodontal risk. The author also calculated personalized supportive periodontal therapy (SPT) interval tools and found that the recommended interval time was three months [21]. Unfortunately, the patient did not stick to the recommended schedule.

A recent trend for the treatment of the periodontal condition is using probiotics, paraprobiotics, and postbiotics as adjunctive therapies for scaling and root planning. Unlike conventional antibiotic therapy, these technologies can positively affect periodontal health with minimum side effects [10, 22]. Probiotics, such as *Lactobacillus reuteri* can modulate the local environment, making SRP efficient [22]. A recent study by Butera et al. found no significant differences in periodontal parameters in the group using postbiotics versus conventional chlorhexidine gel, indicating that postbiotics can be used as a natural alternative to chlorhexidine [22]. Butera et al. also conducted a study to evaluate the efficacy of different kinds of toothpaste containing hyaluronic acid, lactoferrin, and paraprobiotics in reducing gingival bleeding in periodontal patients [23]. Although all kinds of toothpaste show improvement concerning gingival bleeding, paraprobiotics display the most important benefit due to their immunomodulation property [23]. In the future, it is possible that probiotics, paraprobiotics, and postbiotics can be incorporated into the treatment of EPL, especially in phase I (adjunctive to SRP) and phase IV (at-home treatment) periodontal therapy. It is necessary to develop further research to address this issue.

This study has some limitations. First, the follow-up of this patient is done for up to four years. Like any other periodontal therapy, longer follow-up is needed to prove the success of EPL treatment. In addition, this report only displays one case. There is a need to explore more cases regarding the management of EPL, and randomized clinical trials should confirm it.

## 4. Conclusion

The present case shows the importance of understanding the nature of EPL. Comprehensive investigation can guide clinicians in obtaining a proper diagnosis and constructing a final treatment plan. Treatment must adhere to four phases of periodontal therapy, which involve other dental specialties. Clinicians should take note of the interval between treatments as well. Once active treatment is completed, clinicians should emphasize strongly SPT for the long-term success of EPL.

## Figures and Tables

**Table 1:** Periodontal Chart of Tooth 26 and 27.

Visit	Sites	Clinical Measurement	26			27		
			Mesial	Mid	Distal	Mesial	Mid	Distal
First Visit	Buccal	Gingival Recession	2	3	3	2	2	2
		Probing Depth	9	3	6	5	3	2
	Palatal	Probing Depth	14	3	7	4	3	2
		Gingival Recession	2	2	2	2	3	2
After endodontic treatment	Buccal	Gingival Recession	2	2	3	0	2	0
		Probing Depth	2	2	5	4	2	2
	Palatal	Probing Depth	6	2	2	3	2	2
		Gingival Recession	3	3	3	2	3	1
Five months after the periodontal regenerative procedure	Buccal	Gingival Recession	2	3	3	1	1	1
		Probing Depth	3	1	2	1	1	2
	Palatal	Probing Depth	5	3	1	2	2	2
		Gingival Recession	1	1	1	2	2	2
Four-year follow up	Buccal	Gingival Recession	5	5	5	2	2	2
		Probing Depth	2	2	1	1	1	1
	Palatal	Probing Depth	1	2	1	1	1	1
		Gingival Recession	3	2	3	3	5	3

## References

[1] Çirakoğlu N. Y., Karayürek F. (2021). Knowledge and awareness levels of dentists’ about the endo-perio lesions: the questionnaire-based research. *Journal of Health Sciences of Adıyaman University*.

[2] Nainar D. A., Alamelu S., Kv A. (2016). Microbiological profile in endodontic-periodontal lesion. *Journal of Operative Dentistry & Endodontics*.

[3] Papapanou P. N., Sanz M., Buduneli N. (2018). Periodontitis: consensus report of workgroup 2 of the 2017 world workshop on the classification of periodontal and peri-implant diseases and conditions. *Journal of Periodontology*.

[4] Anand V., Govila V., Gulati M. (2012). Endo-perio lesion: part I (the pathogenesis) – a review. *Archives of Dental Sciences*.

[5] Parolia A., Gait T., Porto I., Mala K. (2013). Endo—perio lesion: a dilemma from 19th until 21st century. *Journal of Interdisciplinary Dental*.

[6] Anand V., Govila V., Gulati M. (2012). Endo-perio lesion: part II (the treatment)—a review. *Archives of Dental Sciences*.

[7] Prashaanthi N., Rajasekar A., Shantha Sundari K. K. (2021). Prevalence of endo perio lesion-an institutional study. *International Journal of Dentistry and Oral Science*.

[8] Khasnis S. A., Kidiyoor K. H., Patil A. B., Kenganal S. B. (2014). Vertical root fractures and their management. *Journal of Conservative Dentistry*.

[9] Morales A., Contador R., Bravo J. (2021). Clinical effects of probiotic or azithromycin as an adjunct to scaling and root planning in the treatment of stage III periodontitis: a pilot randomized controlled clinical trial. *BMC Oral Health*.

[10] Scribante A., Gallo S., Pascadopoli M. (2022). Management of periodontal disease with adjunctive therapy with ozone and photobiomodulation (PBM): a randomized clinical trial. *Photonics*.

[11] Bonaccorso A., Tripi T. R. (2014). Endo-perio lesion: diagnosis, prognosis and decision-making. *ENDO*.

[12] Abbott P., Salgado J. (2009). Strategies for the endodontic management of concurrent endodontic and periodontal diseases. *Australian Dental Journal*.

[13] Alshawwa H., Wang J., Liu M., Sun S. (2020). Successful management of a tooth with endodontic-periodontal lesion: a case report. *World Journal of Clinical Cases*.

[14] Fan J., Caton J. G. (2018). Occlusal trauma and excessive occlusal forces: narrative review, case definitions, and diagnostic considerations. *Journal of Periodontology*.

[15] Azouni K. G., Tarakji B. (2014). The trimeric model: a new model of periodontal treatment planning. *Journal of Clinical and Diagnostic Research*.

[16] Bashutski J., Oh T., Chan H., Wang H. (2011). Guided tissue regeneration: a decision-making model. *Journal of the International Academy of Periodontology*.

[17] Wang H.-L., Boyapati L. (2006). ‘PASS’ principles for predictable bone regeneration. *Implant Dentistry*.

[18] Nayer A., Rayyan M., Osman E., Badr S. (2012). An update on the effect of crown margin locations and materials on periodontal health. *Egyptian Dental Journal*.

[19] Farooqi O. A., Wehler C. J., Gibson G., Jurasic M. M., Jones J. A. (2015). Appropriate recall interval for periodontal maintenance: a systematic review. *The Journal of Evidence-Based Dental Practice*.

[20] Lang N. P., Tonetti M. S. (2003). Periodontal risk assessment (PRA) for patients in supportive periodontal therapy (SPT). *Oral Health & Preventive Dentistry*.

[21] Ramseier C. http://www.perio-tools.com.

[22] Butera A., Gallo S., Pascadopoli M., Taccardi D., Scribante A. (2022). Home oral care of periodontal patients using antimicrobial gel with postbiotics, lactoferrin, and aloe barbadensis leaf juice powder vs. conventional chlorhexidine gel: a split-mouth randomized clinical trial. *Antibiotics*.

[23] Butera A., Gallo S., Maiorani C. (2021). Management of gingival bleeding in periodontal patients with domiciliary use of toothpastes containing hyaluronic acid, lactoferrin, or paraprobiotics: a randomized controlled clinical trial. *Applied Sciences*.

